# Multihipposenet: multi-task deep learning for automated DDH assessment in infant hip ultrasound

**DOI:** 10.3389/fped.2026.1939157

**Published:** 2026-10-16

**Authors:** Wenjing Chi, Hong Qi, Huawei Zhao, Hui Cheng, Quanlian Yuan, Shuai Zheng

**Affiliations:** 1 Department of Ultrasound, Haidian District Maternal and Child Health Hospital, Beijing, China; 2 Department of Prenatal Diagnosis, Haidian District Maternal and Child Health Hospital, Beijing, China; 3 Senior Department of Orthopaedics, Fourth Medical Center of PLA General Hospital, Beijing, China; 4 Director of the Department of Child Health and the Division of Comprehensive Early Childhood Development, Haidian District Maternal and Child Health Hospital, Beijing, China

**Keywords:** developmental dysplasia of the hip, Graf ultrasonography, landmark detection, multi-task learning, structure segmentation

## Abstract

**Introduction:**

Developmental dysplasia of the hip (DDH) is a common skeletal condition in infants and young children. For infants under six months, diagnosis relies on Graf ultrasonography to measure the α and β angles, which assess hip-joint development. However, this method requires experienced sonographers with expertise in complex anatomy.

**Methods:**

In this study, we propose an end-to-end multi-task learning network that improves DDH assessment by jointly exploiting complementary anatomical information. First, we use ResNet-50 with a feature pyramid network (FPN) and a contextual semantic fusion module to extract multi-scale features from hip anatomy. Second, we employ a multi-expert gated channel transformation (ME-GCT) unit that explicitly models the relationships between tasks and channels to balance collaboration and conflict across tasks. Finally, a multi-task graph adaptive learning mechanism (MT-GALM) mines the hidden dynamic dependencies between adjacent channels across tasks. Two datasets comprising 1,355 annotated hip ultrasound images from 566 infants were constructed for this work.

**Results:**

Across the patient-level cross-validation test folds, 97.97% and 96.81% of the estimated α and β angles fall within 5 degrees of the expert reference.

**Discussion:**

Because validation was restricted to Graf type I and type II hips, these results should be interpreted as evidence of technical feasibility rather than of established clinical utility; evaluation on type III and type IV hips is required before clinical deployment.

## 1 Introduction

Developmental dysplasia of the hip (DDH) is a prevalent condition in pediatric orthopedics (1), encompassing a spectrum from unstable hips to acetabular or femoral dysplasia, subluxation, and complete dislocation. The incidence among newborns is estimated at 1.5%–2% (2). Early diagnosis and appropriate treatment can restore normal function (3); conversely, untreated DDH may progress to chronic pain, deformity, and early-onset degenerative disease, ultimately resulting in disability (4). Clinical examination by the Barlow and Ortolani maneuvers is the first screening step, but its sensitivity and specificity are limited. For infants under six months, plain radiography involves ionizing radiation (5, 6) and MRI typically requires sedation (7, 8); Graf ultrasonography has therefore become the preferred screening modality because it is non-invasive, radiation-free, and dynamic (9–12). The method classifies hip morphology by measuring the α and β angles in a standard coronal plane (Figure 1): the *α* angle quantifies the bony acetabular socket, and the β angle quantifies the cartilaginous roof.

**Figure 1:**
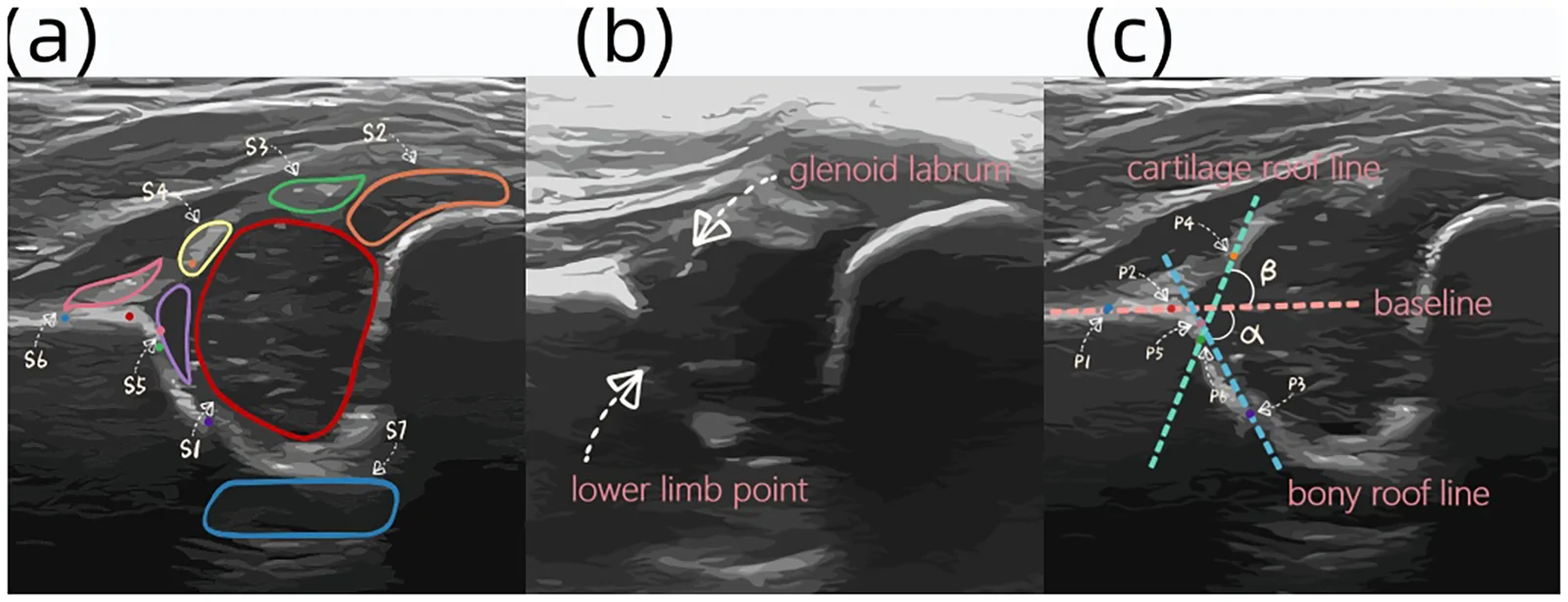
Examples of hip-joint ultrasound images. **(a)** Anatomical structures of the standard plane, showing seven structures S1–S7: S1, femoral head; S2, greater trochanter; S3, glenoid labrum; S4, cartilaginous acetabular roof; S5, synovial fold; S6, proximal perichondrium; S7, Y-type cartilage. **(b)** A non-standard-plane image in which the lower-limb point of the ilium and the labrum are not clearly shown. **(c)** Six landmarks P1–P6 of the standard plane: P1, baseline point (BP); P2, bony-rim point (BRP); P3, lower-limb point (LLP); P4, centre of the labrum point (COP); P5, peak of the acetabular prominence point (POAP); P6, echo-loss point (ELP). All panels are two-dimensional Graf hip ultrasound images. DDH, developmental dysplasia of the hip; α and β are the Graf dysplasia angles defined in the main text.

The reproducibility of Graf ultrasonography depends on measurements taken in the standard plane. As the infant moves and the scanning angle varies, key structures may not be completely visualized (Figure 1B), and diagnostic results on such non-standard images are often unreliable. Despite the popularity of the Graf method, several factors affect assessment consistency, including differences in imaging resolution and quality, inconsistent interpretation (13), and intra- and inter-observer variability (14–17). Even experienced clinicians report standard deviations of up to 3° for the *α* angle and 6° for the β angle, largely because of differences in expertise and image quality (18). Computer-assisted technology can enhance consistency and efficiency (19) by automating the identification of anatomical structures and the measurement of diagnostic metrics, reducing the burden on sonographers and guiding inexperienced clinicians.

Deep-learning research in DDH falls into two main categories: angle measurement and hip-image classification. For angle measurement, landmark coordinates are derived from image features to compute diagnostic metrics. Golan et al. (20) used a convolutional network with adversarial components to segment the ilium and lower limb for automated Graf metrics. Lee et al. (21) combined a two-stage segmentation and landmark-extraction process to measure the α and β angles. Hu et al. (22) proposed a multi-task framework that jointly learns the relationships among landmarks and structures, achieving approximately 93% and 85% of α- and β-angle estimation errors within 5°. Xu et al. (23) developed an end-to-end architecture for more accurate hip-landmark detection, and Chen et al. (24) achieved automatic Graf-type classification approaching human-level performance. Recent studies have further expanded automated DDH assessment through transfer-learning-based detection (25), multicentre validation requirements (26), and geometric radiomic features (27). Despite these advances, most existing methods focus on isolated landmarks, predefined measurements, or image-level classification, making limited use of the multiple anatomical structures needed to verify the standard plane.

Two key limitations thus persist: structural information in hip images is under-utilized, and most methods do not assess the standard plane, risking measurements on inadequate images. Multi-task learning provides a principled approach to these issues by jointly optimizing related objectives so that shared representations capture inter-task dependencies (28). This can be implemented through hard parameter sharing or soft parameter sharing, as in Multi-gate Mixture-of-Experts (29), progressive layered extraction (30), and unified feature optimization (31). In medical imaging, jointly predicting landmarks and segmenting structures has been shown to capture latent anatomical correlations and improve both tasks (32–36). This paradigm is well suited to DDH diagnosis, where structure boundaries and landmark positions are inherently coupled.

This study explicitly considers the interrelations among hip-joint structures and landmarks and introduces a multi-task learning network, MultiHipPoseNet, for the measurement and diagnosis of DDH. The primary contributions of this paper are as follows:

1. A multi-task framework that utilizes nearly all hip-imaging information, including seven structures and six landmarks, and integrates structural segmentation with landmark detection, providing a more comprehensive diagnostic basis for DDH.
2. A multi-expert gated channel transformation (ME-GCT) unit that optimizes feature extraction by explicitly modelling the relationships between tasks and channels to balance collaboration and conflict across tasks.
3. A multi-task graph adaptive learning mechanism (MT-GALM) that mines the hidden dynamic dependencies between adjacent channels across tasks, reducing the risk of overfitting to a single task and improving robustness.

## 2 Methods

### 2.1 Framework overview

The overall workflow of the proposed MultiHipPoseNet framework is illustrated in Figure 2. Given a hip ultrasound image, the network first extracts multi-scale representations and then adaptively refines task-related channel features through ME-GCT. The feature maps from the structure and landmark branches are further combined with low-level spatial features and processed by MT-GALM to capture cross-task dependencies. The framework finally outputs seven anatomical masks and six landmark predictions, which are used for DDH measurement and analysis.

**Figure 2:**
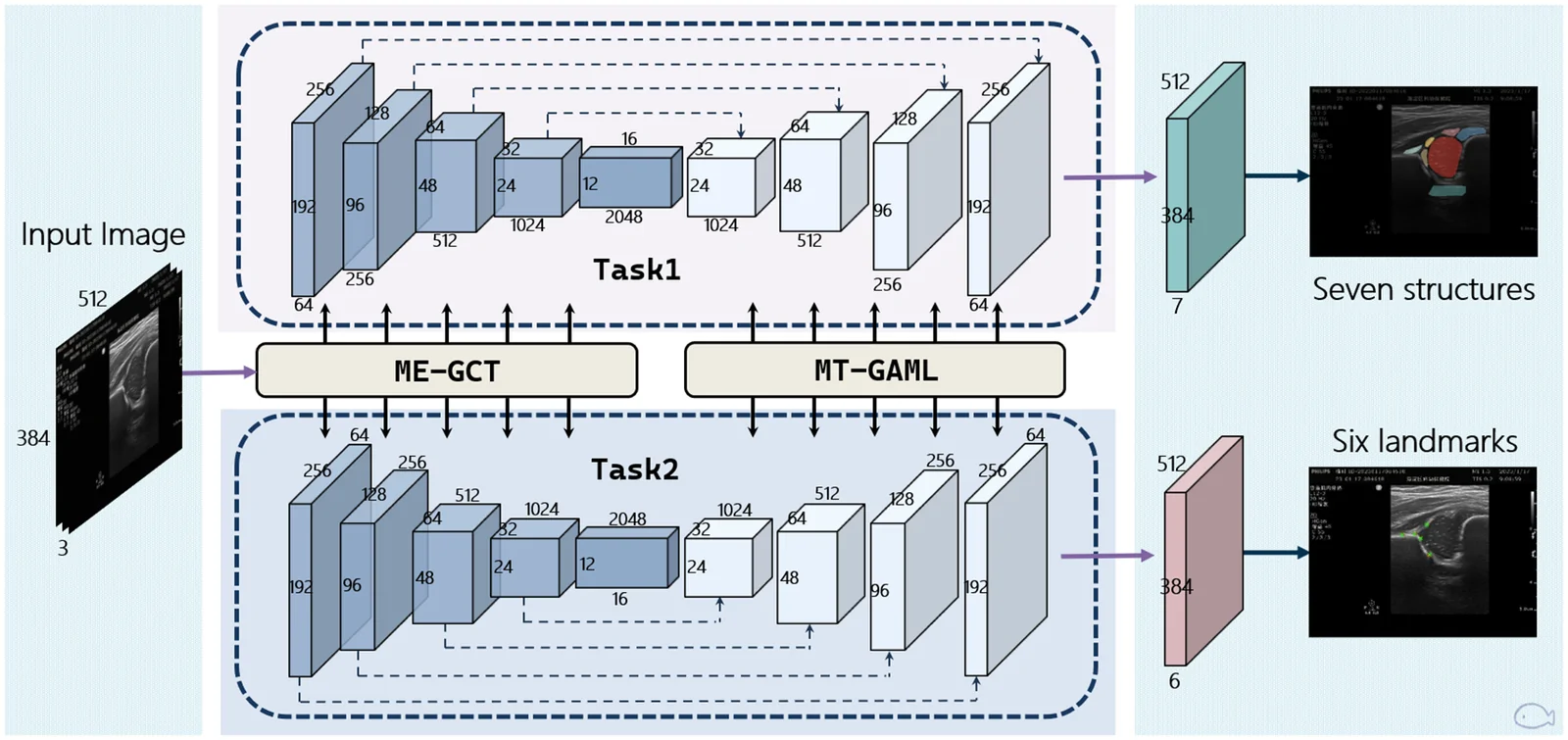
Schematic of the multiHipPoseNet algorithm. ME-GCT, the multi-expert gated channel transformation unit, models the relationships between tasks and channels by combining multiple experts' channel weights. MT-GALM, the multi-task graph adaptive learning mechanism, mines the hidden dynamic dependencies between adjacent channels across tasks. For visual clarity, the main schematic depicts the task streams and a single ME-GCT block; the three expert branches (m = 3) are expanded in Figure 4. The framework outputs seven anatomical-structure masks and six landmark heatmaps, which are used to compute the Graf α and β angles. FPN, feature pyramid network.

### 2.2 Multi-task learning network

To exploit the complementary spatial relationships between anatomical structures and keypoints, we design a unified multi-task framework that simultaneously performs (i) detection and segmentation of multiple anatomical structures and (ii) localization of multiple landmarks. Accurate DDH assessment requires robust feature representation from hip ultrasound images, especially for weak boundaries and small anatomical targets. Previous landmark-aware studies have jointly performed anatomical-structure segmentation and landmark digitization (32), used automatically identified landmarks to extract disease-related features from brain MR images (33–35), and modelled spatial relationships among landmarks using graph convolution (36). These studies demonstrate the value of anatomically informed feature learning; however, they were developed for CBCT or brain MR images and do not directly address the weak boundaries, low contrast, and speckle noise of infant hip ultrasonography.

The core rationale of our multi-task design is that jointly learning structure segmentation and landmark localization allows the network to capture latent anatomical correlations, thereby improving both sub-tasks. Popular segmentation and landmark-detection networks such as U-Net (37), FCN (38), and ResNet (39) have been used on hip ultrasound images. Hu et al. (22) introduced a multi-task framework for DDH based on Mask R-CNN (40), in which anatomical structures and landmarks are learned jointly. In our method, ResNet-50-FPN encodes hierarchical features, and U-Net-like feature fusion preserves fine spatial information for anatomical boundaries and landmarks. Here, the contextual semantic fusion module mentioned in the Abstract refers to the top-down FPN pathway augmented with a lightweight context block: the highest-level feature map is passed through parallel dilated convolutions (dilation rates 1, 3, and 5) and fused with each up-sampled pyramid level by element-wise addition, so that global anatomical context is propagated to the high-resolution levels used for boundary and landmark prediction. As shown in Figure 3, the network contains two prediction heads that produce seven structure masks and six landmark heatmaps, respectively.

**Figure 3:**
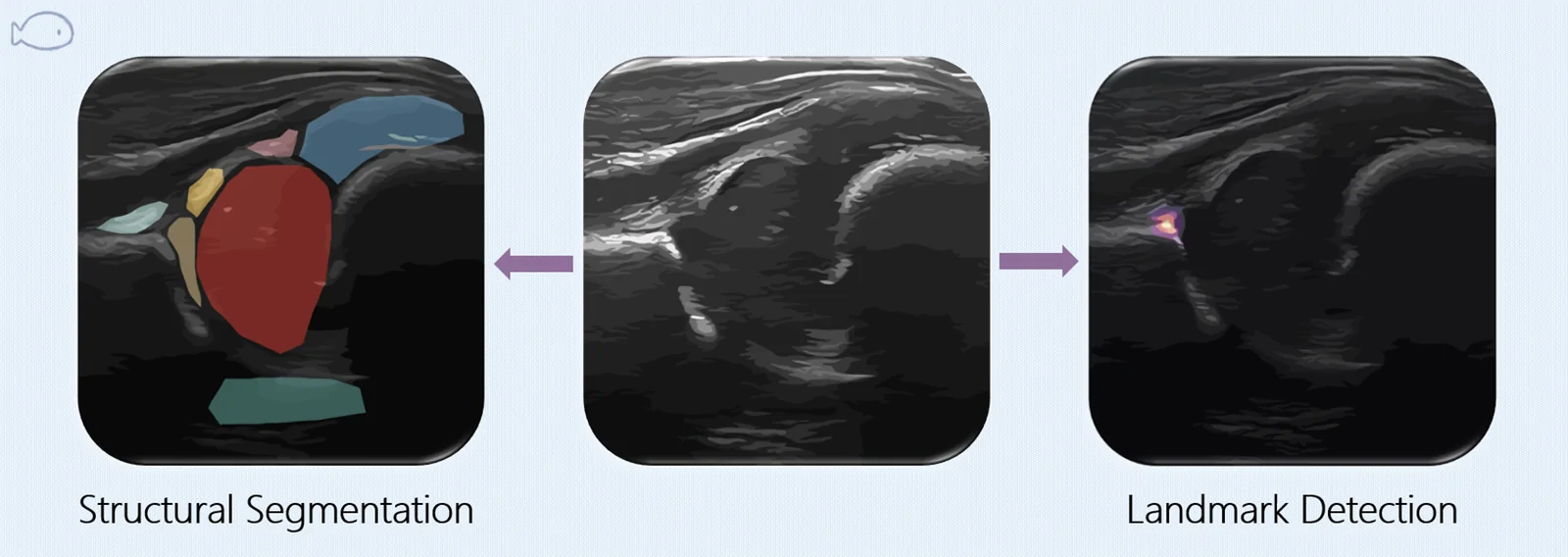
Task generation. For structural segmentation, a segmentation mask is established for each region of interest. For landmark detection, the task is cast as regression of a heatmap representing the local neighbourhood of each landmark. In the right diagram, purple to tan denotes values from zero to one. Left, a segmentation mask per anatomical region of interest; right, a Gaussian heatmap regression target per landmark.

To reduce task imbalance and alleviate gradient conflict, the loss weights are updated dynamically according to the coefficient of variation of the task losses during training (41). The overall training objective is a dynamically weighted sum of the structure and landmark losses (Equations 1). The normalized coefficient-of-variation weights are defined in Equations 2, where cᵥᵢᵗ is the coefficient of variation of the loss of task i at training step t (its running standard deviation divided by its running mean), and zᵗ is the normalizing constant. This normalization ensures that the task weights sum to one and decouples loss weighting from the learning rate. The structure loss combines a focal loss and a Dice loss (42, 43), and the landmark loss is a distance-weighted mean-squared-error loss (44), as defined in Equations 3, 4. In the focal loss, pₜ denotes the predicted probability of the ground-truth class (pₜ = *p* if the structure label yₛ = 1 and pₜ = 1−*p* otherwise), *γ* is the focusing parameter that down-weights easy pixels, and *κ* is a class-balancing weight that compensates for the small size of thin structures. In the landmark loss, ŷₗ and yₗ are the predicted and ground-truth heatmaps, and *λ* is a hyperparameter that exponentially weights the ground-truth heatmap so that the penalty is largest at the landmark and decreases to 1 as the distance from the landmark increases. In Equations 3, 4, *p* indexes pixels, *Ω* is the image domain, and *ε* is a small constant that prevents division by zero. All symbols (*κ*, *γ*, *λ*, and *δ*) are thus defined explicitly.

$$L\;=\;\delta_{s}^{t\;}\;L\_struct\;+\;\delta_{l}^{t}\;L\_land\;$$ (1)

$$\delta_{i}^{t\;}\;=\;\;cv_{i}^{t}\;/\;Z^{t},\;\;\;Z^{t}\;=\;\sum_{i}\;cv_{i}^{t},\;\;\;\sum_{i}\;\delta_{i}^{t}\;=\;1$$ (2)

$$L\_struct\;=\;-\;\kappa {\left(1\;-\;p_{t}\right)}^{\gamma }\;ln\left(\;p_{t}\right)\;+\;1\;-\;\left[2\sum_{p\;}\;{\hat{y}}_{s}\;\left(p\right)\;y_{s}\left(p\right)\;+\;\varepsilon \right]/\left[\sum_{p\;}\;{\hat{y}}_{s}\;{\left(p\right)}^{2}\;+\;\sum_{p}\;y_{s}\left(p\right){\;}^{2}+\;\varepsilon \right]$$ (3)

$$L\_land\;=\;|\Omega {|}^{-1}\;\sum_{p}\;\in \;\Omega \;\lambda {\;}^{\wedge }\;\left[y1\;\left(p\right)\right]\;{\left[\hat{y}1\;\left(p\right)-\;y1\;\left(p\right)\right]}^{2}$$ (4)

### 2.3 ME-GCT

Although multi-task learning can improve representation sharing, inappropriate parameter sharing may cause negative transfer and degrade individual-task performance (45, 46). Many multi-task models are sensitive to data-distribution differences and task relationships (47, 48). Conflicts arising from task disparities can harm at least some tasks, particularly when parameters are extensively shared. In shallow layers, the model mainly learns low-level features such as textures and edges, which require channel cooperation for broad extraction; in deeper layers, the focus shifts to high-level, task-specific features that require channel competition for valuable information. Task relationships and channel-level interactions should therefore be modelled explicitly to enable selective knowledge sharing.

Inspired by Multi-gate Mixture-of-Experts (29), we design ME-GCT to perform task-adaptive channel selection from multiple expert feature extractors. As shown in Figure 4, each expert network, ResNet-50-FPN, is a feed-forward network. We adapt the gated channel transformation unit (49) as the gating network to process input features, combining experts with varying channel weights so that distinct tasks leverage the experts differently. The results are passed to task-specific sub-networks, enabling each task to learn diverse expert-assembly patterns and to capture task and channel relationships. As defined in Equations 5, the output yₖ of task k is a weighted sum over expert channels, where the gate gₖ produces a distribution over the experts' channels and the outputs of all experts' channels are combined accordingly.

$$y_{k}=\overset{m}{\underset{i\;=\;1}{\sum }}\;g_{k}{\left(x\right)}_{i}e\;f_{i}\left(x\right),\overset{m}{\underset{i\;=\;1}{\sum }}\;g_{k}{\left(x\right)}_{i}=1$$ (5)

**Figure 4:**
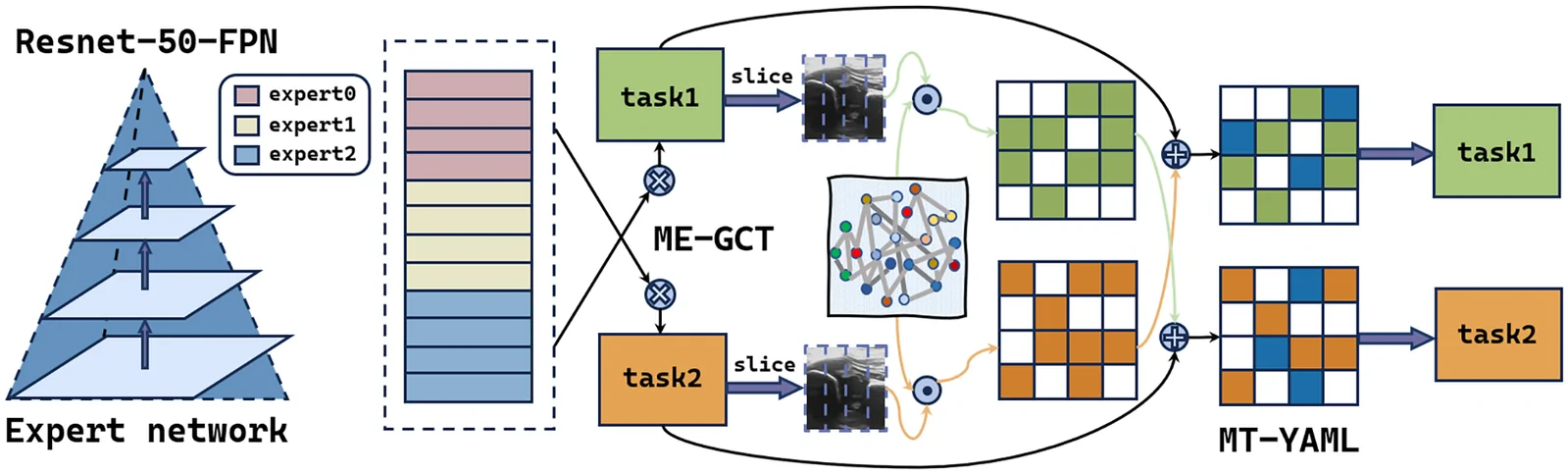
Illustration of ME-GCT and MT-GALM. ⊗ denotes the weighted combination of multi-expert channels, and ⊕ denotes the addition of task features and information-gain features. For task 1, the initial task features are generated by combining multi-expert channel weights; the feature slices, represented as nodes, are processed by a graph neural network to extract the information-gain features relevant to task 1 (green) from other tasks (blue), yielding features that capture both task 1 and its implicit dependencies with other tasks. The same procedure is applied to task 2. Three expert branches (m = 3) are shown. ME-GCT, multi-expert gated channel transformation; MT-GALM, multi-task graph adaptive learning mechanism.

Each gate learns input-dependent expert-channel weights, allowing tasks to use shared representations flexibly and task-specifically. ME-GCT captures task relationships by determining how the separations created by different gates overlap. When tasks are less related, expert sharing is penalized, prompting the gating networks to use different experts. In a special case of Mixture-of-Experts, if only the highest-scoring expert is chosen, each gate linearly segments the input space into m regions, one per expert. In contrast, ME-GCT divides the input space into m · n regions corresponding to the experts' channel features, adding only a few gating parameters, which are negligible. It can thus explicitly model relationships between tasks and channels through interpretable control variables while retaining the benefits of knowledge transfer.

### 2.4 MT-GALM

Graph neural networks provide an effective way to represent and propagate dependencies among non-Euclidean feature nodes (50). Through neighbourhood aggregation, each node's representation is updated using information from connected nodes. In our framework, each image-feature channel is treated as a graph node, and latent dependencies are learned across the structure-segmentation and landmark-localization branches. Because the two tasks differ in their supervision forms and feature requirements, direct hard parameter sharing may introduce interference. We therefore designed MT-GALM to learn task-specific channel dependencies and selectively incorporate complementary information from the other branch. The general graph-neural-network principle follows (50), whereas the cross-task channel graph and feature-fusion strategy were developed for MultiHipPoseNet.

MT-GALM learns adaptive graph connections among task-related feature channels, enabling information exchange across branches and improving robustness. As shown in Figure 4, for a given task (e.g., task 1), feature channels from related branches are regarded as graph nodes and used to construct a task-specific dependency graph. We then learn information-gain features from the other tasks and fuse them with the target-task features, producing refined representations that preserve task-specific cues while incorporating complementary cross-task information. Formally, a graph in each layer is G = (V, E), where V is the set of nodes corresponding to image-feature channels and E is the set of edges. The adjacency matrix is Aᵥ ∈ R^{N × N}; for nodes vᵢ, v_j_ ∈ V, (Aᵥ)ᵢ,_j_ is one if (vᵢ, v_j_) ∈ E and zero otherwise. Because image-feature channels have no predefined graph topology, we adopt the learnable graph-construction principle of Liu et al. (51) to generate adaptive adjacency matrices among feature dimensions. Whereas TodyNet (51) models hidden dependencies among variables in multivariate time series, our framework applies this principle to cross-task image-feature channels and jointly optimizes the learned adjacency matrices with the two branches. We randomly initialize two node-embedding dictionaries with learnable parameters E₁, E₂ ∈ R^{N × c}; the self-adaptive adjacency matrix is defined in Equations 6.

$$A\;=\;SoftMax\left[ReLU\;\left(E_{1}E_{2}^{T}\right)\right]$$ (6)

Here E₁ is the source-node embedding and E₂ is the target-node embedding. Multiplying E₁ and E₂ yields the spatial-dependency weights between source and target nodes. The ReLU activation removes weak connections and the SoftMax normalizes the self-adaptive adjacency matrix, which serves as the final adjacency matrix. Through graph adaptive learning, we introduce the graph-convolution update in Equations 7 for task k′, in which Z is the output, X the input features, W the parameter matrix, Ã = A + I is the adjacency matrix with self-connections, D is its degree matrix, *σ* is the activation function, and the transformed message is fused with the target-task feature.

$$Z_{k}=\sigma {\left(D^{:-1}{/}^{2}A^{:}D^{:-1}{/}^{2}XW\right)}_{k},A^{:}=A+I$$ (7)

### 2.5 Implementation details

All models were implemented in PyTorch and trained on a single NVIDIA GeForce RTX 3090 GPU (24 GB). Input images were resized to 512 × 384 pixels, consistent with Figure 2. The ResNet-50-FPN backbone was initialized with ImageNet-pretrained weights, and the ME-GCT gates, graph modules, and prediction heads were initialized with Xavier initialization. The network was optimized with Adam (β₁ = 0.9, β₂ = 0.999, weight decay 1 × 10⁻⁴) using an initial learning rate of 1 × 10⁻⁴ decayed by a cosine schedule with a 5-epoch linear warm-up, a batch size of 8, and 200 training epochs with early stopping on the validation loss (patience 20). Data augmentation comprised random horizontal flipping, rotation within ±10°, random scaling of 0.9–1.1, gamma adjustment, and additive speckle noise to emulate ultrasound artefacts. In ME-GCT, the number of experts was m = 3 and the gating network followed the gated channel transformation unit (49). In MT-GALM, each cross-task graph used *N* = 256 channel nodes with a node-embedding dimension of c = 16 and a single graph-convolution layer per task. The focusing parameter of the focal loss was *γ* = 2; *κ* was the normalized inverse-frequency weight for foreground versus background pixels calculated from the training partition; the heatmap weighting factor was *λ* = 5; and *ε* was 1 × 10⁻⁶.

Landmark coordinates and diagnostic angles were obtained by a deterministic post-processing step. For each of the six landmark heatmaps, the pixel with the maximum response was located and refined to subpixel accuracy by a local centre-of-mass over a 5 × 5 window, giving the landmark coordinate. The baseline was fitted through the baseline and bony-rim points, the bony-roof line through the lower-limb and bony-rim points, and the cartilage-roof line through the echo-loss and labral-centre points; each line was obtained by total least-squares fitting. The *α* angle was computed as the angle between the baseline and the bony-roof line and the β angle as the angle between the baseline and the cartilage-roof line. No manual cropping or interactive correction was used at any stage, so the pipeline is fully automatic.

### 2.6 Datasets and ethics

Two retrospective datasets of anonymized two-dimensional Graf hip ultrasound images were constructed from two ultrasound machines at a single institution. Dataset A (machine 1) was used for model development, with both hips imaged and one to three standard-plane frames retained per hip; Dataset B (machine 2) was reserved as an independent external test set that was not seen during training. Each image was annotated by experienced pediatric sonographers with seven anatomical structures and six landmarks. To prevent information leakage, all data were split at the patient (infant) level rather than the image level, so that every image from a given infant appeared in exactly one of the training, validation, or test partitions. For Dataset A we used five-fold patient-level cross-validation (approximately 70% training, 10% validation, and 20% test per fold). Because the cohort comprised predominantly Graf type I hips, with fewer type II hips and no type III or type IV (frankly dislocated) hips, the two available types were trained together using class-balanced mini-batch sampling and the focal loss described in Section 2.2, and test results were reported separately for each type; the resulting cohort composition is reported in Section 3.1. Because type III and type IV hips were absent, the evaluation characterizes performance on normal and mildly dysplastic hips only.

This retrospective study was conducted in accordance with the Declaration of Helsinki and was approved by the Ethics Committee of Haidian District Maternal and Child Health Hospital (approval No. 2024-15). All ultrasound examinations were clinically indicated and performed as part of routine Graf screening for DDH during standard clinical care; no imaging was acquired prospectively for research purposes, and Dataset A and Dataset B differ only in the ultrasound machine used. General consent for the diagnostic examination was obtained from the legal guardians as part of routine care at the time of imaging. Because the present work is a retrospective secondary analysis of previously acquired, fully anonymized diagnostic images, the Ethics Committee approved this secondary use and waived the requirement for study-specific informed consent. For the same reason—this being a retrospective analysis rather than a prospective or interventional trial—clinical-trial registration is not applicable.

### 2.7 Statistical analysis

Landmark error, structure-segmentation metrics, and angle error were first calculated at the image level. To avoid treating multiple frames from the same infant as independent observations, inferential comparisons were performed on infant-level means. Paired method comparisons used a two-sided paired t-test when the distribution of within-infant differences was approximately normal; otherwise, the Wilcoxon signed-rank test was used. Predicted and reference angles were assessed with Pearson's correlation coefficient, Bland–Altman bias and 95% limits of agreement (mean difference ± 1.96 standard deviations), and a two-sided paired t-test of the prediction errors against zero. All tests were two-sided, and *p* < 0.05 was considered statistically significant. Because the type II subgroup was small (its composition is reported in Section 3.1), type-specific metrics were treated as descriptive and no formal type I-versus-type II hypothesis test was used.

Landmark localization was summarized by horizontal error (*Δ*X), vertical error (*Δ*Y), Euclidean error (*Δ*D), and successful detection rate (SDR) within prespecified radial thresholds. Structure segmentation was summarized by the Dice similarity coefficient (DSC), Hausdorff distance (HD) (52), and mean intersection-over-union (MIoU). Image noise was quantified by peak signal-to-noise ratio (PSNR). For type I/type II classification, accuracy, precision, and recall were calculated from the automated Graf category. An angle estimate was prespecified as successful when its absolute error was below 5°. This threshold lies within the previously reported inter-observer variability of experienced sonographers [standard deviations up to 3° for *α* and 6° for β (18)] and was used as a tolerance criterion, not as evidence of equivalence to a clinician. Automated Graf classification used α ≥ 60° and β < 77° for type I and α < 60° for type II.

Robustness was evaluated by applying prespecified levels of salt-and-pepper noise [a common ultrasound artefact (53)] to the Dataset A test images and comparing MultiHipPoseNet with MultiHipPoseNet− at peak signal-to-noise ratios (PSNRs) of 63.043, 43.533, 33.503, 26.526, 23.531, and 20.542 dB; MIoU summarized segmentation performance in this experiment. For the image-quality analysis, the prespecified categories were good (anatomical structures and interfaces clearly visible), medium (blurred structure edges), and poor (one or more target structures poorly visible). Performance was additionally summarized separately for Graf type I and type II images and on the held-out Dataset B from machine 2. No model parameters were updated in the quality-stratified, type-stratified, noise-robustness, or Dataset B analyses.

## 3 Results

### 3.1 Study datasets and experimental setup

The two datasets comprised 1,355 annotated two-dimensional Graf hip ultrasound images from 566 infants. Dataset A (machine 1) contained 1,129 images from 470 infants and was used for five-fold patient-level cross-validation, so that each test fold contained approximately 226 images (200 Graf type I and 26 type II); Dataset B (machine 2) contained 226 images from 96 infants and served only as the external test set. No Graf type III or type IV hips were available in either dataset.

To assess the efficacy of the proposed MultiHipPoseNet, we conduct comparative and ablation experiments against state-of-the-art methods. In all experiments, “Deeplabv3+”, “PSnet”, and “U-Net” denote the segmentation methods of (37, 54, 55), and “U-Net”, “Hrnet-32”, and “Hrnet-48” denote the landmark-detection methods of (37, 56). PSnet, DeepLabv3+, U-Net, HRNet-32, HRNet-48, HipPoseNet, and MultiHipPoseNet− were trained and tested on Dataset A under the protocol described in Section 2.5. By contrast, the methods of Hu et al. (22), Xu et al. (23), and Chen et al. (24) could not be faithfully re-implemented because neither complete code nor trained weights were publicly available; their published values are included only for contextual comparison and are marked with a dagger (†). “MultiHipPoseNet” denotes our full multi-task model; “HipPoseNet” denotes a single-task variant without multi-task learning; and “MultiHipPoseNet−” denotes a multi-task variant that adds ME-GCT but omits MT-GALM. In the tables, bold font indicates the best result, and an asterisk (*) indicates a statistically significant difference in the mean relative to our method. Additional details of the experimental evaluation are provided in the Supplementary Material.

### 3.2 Landmark detection and structure segmentation

For landmark detection, the SDR within 1 mm was 83.08% for MultiHipPoseNet, the highest value among the methods re-implemented on Dataset A (Table 1). At the six landmarks, the *Δ*D values were 1.371, 0.589, 0.604, 0.737, 0.826, and 0.552 mm. The SDR curves indicate that approximately 90% of BRP, LLP, and ELP predictions were within 1.1 mm, whereas approximately 90% of COP and POAP predictions were within 1.4 mm (Figure 5A).

**Table 1:** Landmark-detection results for different state-of-the-art methods.

Method		L1 (BP)	L2 (BRP)	L3 (LLP)	L4 (COP)	L5 (POAP)	L6 (ELP)	SDR 0.5mm	SDR 1mm	SDR 1.5mm
Hrnet-32	*Δ*X	1.486	0.449	0.423	0.631	0.649	0.595			
	*Δ*Y	0.411	0.641	0.646	0.714	0.781	0.438	24.25%	63.50%	87.33%
	*Δ*D	1.620	0.850	0.838	1.043	1.103	0.813			
Hrnet-48	*Δ*X	1.413	0.466	0.441	0.637	0.638	0.633			
	*Δ*Y	0.453	0.632	0.681	0.701	0.772	0.495	22.43%	62.12%	86.21%
	*Δ*D	1.573	0.852	0.874	1.038	1.085	0.887			
Chen et al. (24)^a^	*Δ*X	1.323	0.371	0.352	0.496	0.493	0.472			
	*Δ*Y	0.293	0.527	0.580	0.547	0.587	0.312	36.37%	77.01%	92.92%
	*Δ*D	1.402	0.696	0.719	0.811	0.834	0.623			
HipPoseNet	*Δ*X	1.359	0.376	0.377	0.489	0.547	0.476			
	*Δ*Y	0.290	0.502	0.523	0.562	0.692	0.336	35.93%	76.97%	93.50%
	*Δ*D	1.435	0.686	0.697	0.817	0.948	0.642			
MultiHipPoseNet−	*Δ*X	**1**.**297**	0.302	0.299	0.443	0.495	0.435			
	*Δ*Y	0.249	0.503	0.512	**0**.**522**	0.611	0.264	41.79%	81.01%	94.50%
	*Δ*D	**1**.**356**	0.635	0.632	0.745	0.857	0.559			
MultiHipPoseNet	*Δ*X	1.314	**0**.**287**	**0**.**291**	**0**.**437**	**0**.**463**	**0**.**432**			
	*Δ*Y	**0**.**244**	**0**.**465**	**0**.**486**	0.524	**0**.**604**	**0**.**262**	**44**.**35%**	**83**.**08%**	**94**.**99%**
	*Δ*D	1.371	**0**.**589**	**0**.**604**	**0**.**737**	**0**.**826**	**0**.**552**			

Landmarks 1–6 are BP, BRP, LLP, COP, POAP, and ELP, respectively (Figure 1C). *Δ*X, *Δ*Y, and *Δ*D are the horizontal, vertical, and Euclidean errors (mm); SDR is the successful detection rate within 0.5, 1, and 1.5 mm. ^a^Published values shown for context only; not a like-for-like comparison. Bold indicates the best result among methods evaluated on Dataset A. Values are means over the five Dataset A patient-level cross-validation test folds. α and β are Graf angles; *Δ*X, *Δ*Y, and *Δ*D are horizontal, vertical, and Euclidean landmark errors (mm); SDR is reported within 0.5, 1, and 1.5 mm.

**Figure 5:**
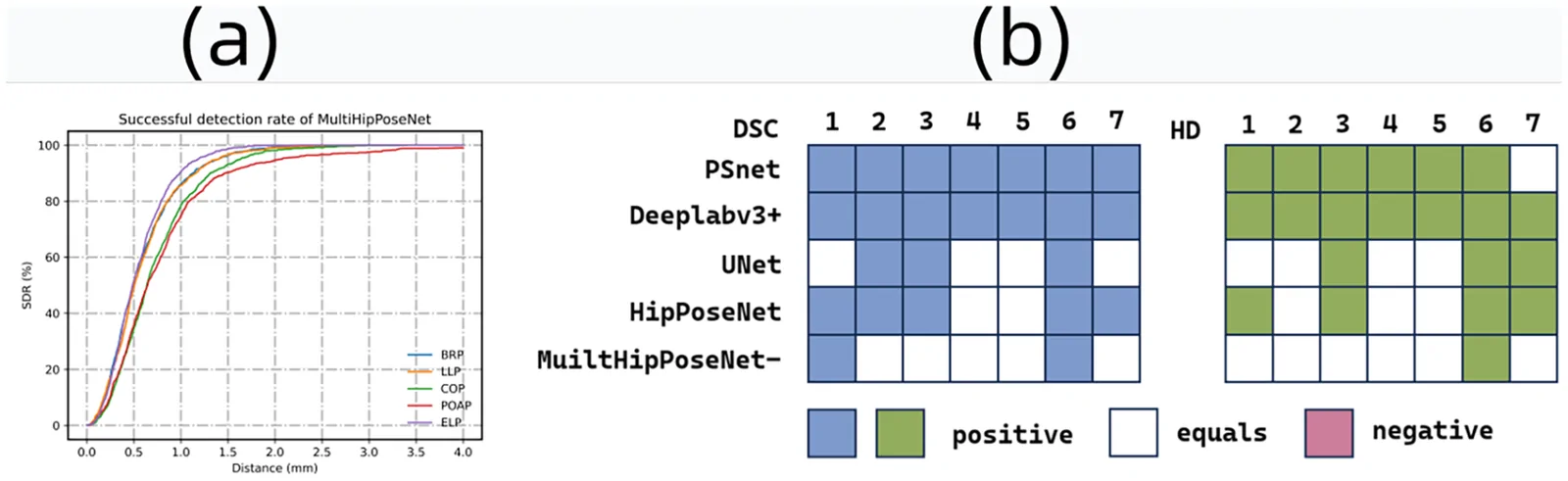
**(a)** successful detection rates of multiHipPoseNet across landmarks. **(b)** Whether other methods differ significantly from MultiHipPoseNet: blue or green indicates *p* ≤ 0.05 with DSC or HD worse than MultiHipPoseNet; white indicates *p* > 0.05 (no significant difference); pink indicates *p* ≤ 0.05 with DSC or HD better than MultiHipPoseNet. SDR, successful detection rate (fraction of predicted landmarks within a given radial distance of the expert reference), evaluated on the Dataset A test folds; DSC, Dice similarity coefficient; HD, Hausdorff distance. Significance in **(b)** is from two-sided paired comparisons of infant-level means (paired t-test, or Wilcoxon signed-rank test when non-normal; *p* ≤ 0.05). BP, BRP, LLP, COP, POAP, and ELP are the six landmarks defined in Figure 1C.

For structural segmentation, the DSC values of MultiHipPoseNet across the seven structures ranged from 0.819 to 0.947 (Table 2), and the corresponding HD values ranged from 4.599 to 14.362 pixels (Table 3). The paired comparisons with MultiHipPoseNet are summarized in Figure 5B. Relative to U-Net, the proposed model had higher DSC and lower HD for the glenoid labrum, and the remaining non-white cells indicate the structure-method comparisons with *p* ≤ 0.05. Two representative concordant predictions and one case with a disrupted cartilage mask are shown in Figure 6.

**Table 2:** Overall segmentation of seven anatomical structures by the dice similarity coefficient (DSC).

Method	Femoral head	Greater trochanter	Glenoid labrum	Cartilaginous acetabular roof	Synovial fold	Proximal perichondrium	Y-type cartilage
PSnet	0.935 (0.003*)	0.866 (0.004*)	0.753 (0.005*)	0.804 (0.002*)	0.774 (0.004*)	0.687 (0.002*)	0.805 (0.012*)
Deeplabv3+	0.938 (0.002*)	0.872 (0.004*)	0.815 (0.003*)	0.847 (0.002*)	0.803 (0.005*)	0.817 (0.006*)	0.811 (0.007*)
U-Net	**0.947** (**0.001)**	0.885 (0.001*)	0.829 (0.002*)	**0.862** (**0.001)**	**0.830** (**0.001)**	0.841 (0.002*)	0.818 (0.004)
HipPoseNet	0.940 (0.002*)	0.885 (0.003*)	0.829 (0.005*)	0.860 (0.003)	0.823 (0.007)	0.830 (0.004*)	0.807 (0.008*)
MultiHipPoseNet−	0.943 (0.002*)	0.888 (0.003)	0.840 (0.005)	0.855 (0.002)	0.828 (0.005)	0.839 (0.004*)	**0.824** (**0.002)**
MultiHipPoseNet	**0.947** (**0.002)**	**0.889** (**0.001)**	**0.842** (**0.005)**	0.859 (0.006)	0.827 (0.007)	**0.846** (**0.002)**	0.819 (0.004)

Numbers in parentheses are standard deviations; an asterisk (*) denotes a significant difference relative to MultiHipPoseNet. Bold indicates the best result. DSC, Dice similarity coefficient (range 0–1; higher is better). Values are the mean (standard deviation) over the five Dataset A test folds for each of the seven anatomical structures; the asterisk denotes a significant difference from MultiHipPoseNet (two-sided paired test of infant-level means, *p* < 0.05).

**Table 3:** Edge accuracy of seven anatomical structures by the hausdorff distance (HD, in pixels).

Method	Femoral head	Greater trochanter	Glenoid labrum	Cartilaginous acetabular roof	Synovial fold	Proximal perichondrium	Y-type cartilage
PSnet	10.922 (0.408*)	12.131 (0.639*)	8.044 (1.950*)	7.160 (0.044*)	9.664 (0.143*)	10.885 (0.471*)	15.194 (1.181)
Deeplabv3+	10.701 (0.313*)	12.251 (0.363*)	5.580 (0.106*)	5.848 (0.297*)	8.906 (0.242*)	5.930 (0.222*)	15.274 (0.799*)
U-Net	**9.291** (**0.256)**	11.032 (0.196)	5.090 (0.095*)	5.127 (0.290)	**7.624** (**0.131)**	5.363 (0.180*)	15.221 (0.544*)
HipPoseNet	10.418 (0.411*)	11.245 (0.338)	5.156 (0.208*)	**5.095** (**0.084)**	8.715 (2.395)	6.020 (0.264*)	15.678 (0.748*)
MultiHipPoseNet−	9.751 (0.366)	**10.556** (**0.318)**	4.765 (0.265)	5.483 (0.394)	7.702 (0.267)	5.298 (0.567*)	14.562 (0.427)
MultiHipPoseNet	9.552 (0.307)	10.816 (0.453)	**4.668** (**0.223)**	5.329 (0.409)	7.916 (0.285)	**4.599** (**0.250)**	**14.362** (**0.533)**

Numbers in parentheses are standard deviations; an asterisk (*) denotes a significant difference relative to MultiHipPoseNet. Bold indicates the best result. HD, Hausdorff distance in pixels (lower is better). Values are the mean (standard deviation) over the five Dataset A test folds; the asterisk denotes a significant difference from MultiHipPoseNet (two-sided paired test of infant-level means, *p* < 0.05).

**Figure 6:**
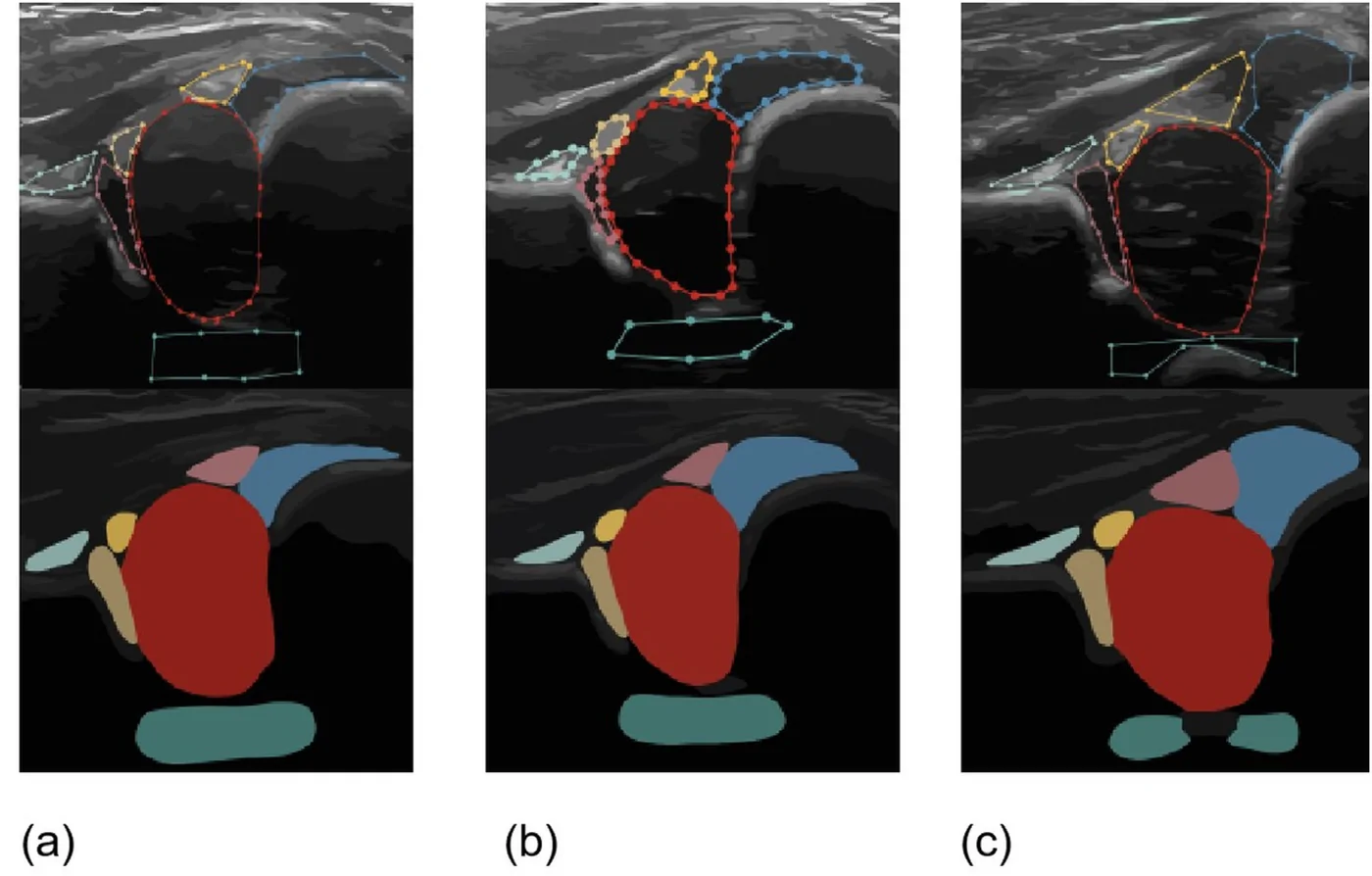
Comparison of multiHipPoseNet with the ground truth. Shown are images annotated by clinicians and the corresponding predictions. **(a)** and **(b)** are predictions under normal conditions; **(c)** shows a ruptured-cartilage image. Coloured overlays denote the seven segmented anatomical structures; all panels are Dataset A test images.

In the ablation comparison (Tables 1–3), MultiHipPoseNet had a higher SDR at 0.5, 1.0, and 1.5 mm than HipPoseNet and MultiHipPoseNet−. For segmentation, the full model had higher DSC than HipPoseNet in six of seven structures and lower HD than MultiHipPoseNet− in five of seven structures.

### 3.3 DDH metric analysis and image classification

The angle errors and Graf type I/type II classification metrics are reported in Table 4. HipPoseNet, MultiHipPoseNet−, and MultiHipPoseNet were trained and tested under the same protocol. The published values of Hu et al. (22), Xu et al. (23), and Chen et al. (24) are flagged with a dagger (†) and are shown only for context because they were obtained on different datasets and are not like-for-like comparisons.

**Table 4:** Comparison of multiHipPoseNet with three advanced methods and its own ablations for DDH analysis.

Method	α (°)	β (°)	Accuracy	Precision	Recall
Hu et al. (22)†	2.221	2.899	94.33%	72.71%	90.71%
Xu et al. (23)†	2.320	2.360	96.20%	73.91%	81.00%
Chen et al. (24)†	1.856	2.081	96.90%	85.60%	88.46%
HipPoseNet	2.032	2.196	96.02%	81.40%	87.69%
MultiHipPoseNet−	1.695	1.802	97.26%	91.65%	83.85%
MultiHipPoseNet	**1**.**662**	**1**.**756**	**97**.**79%**	**92**.**11%**	**88**.**46%**

α and β are mean absolute angle errors (°); accuracy, precision, and recall refer to type I/type II classification. A dagger (†) marks values reproduced from the original publication because code and weights were not publicly available; these rows are contextual and not like-for-like. Bold indicates the best result among methods evaluated on Dataset A. α and β are mean absolute angle errors (degrees; lower is better); accuracy, precision, and recall (%) refer to automated Graf type I/type II classification. Values for methods evaluated here are means over the five Dataset A test folds (200 type I and 26 type II images per fold).

MultiHipPoseNet achieved mean absolute errors of 1.662° for *α* and 1.756° for β. On Dataset A, 97.97% and 96.81% of the α- and β-angle estimates, respectively, had an absolute error below 5°. Scatter and Bland–Altman plots of the predicted and true α and β angles are shown in Figure 7. The Pearson correlation coefficients were 0.92 for *α* and 0.91 for β. The Bland–Altman bias was −0.11° for *α* [95% limits of agreement (LoA), −4.14° to 3.92°] and −0.17° for β (95% LoA, −4.15° to 3.80°). Paired t-tests of the prediction errors against zero gave *p* = 0.66 for *α* and *p* = 0.43 for β.

**Figure 7:**
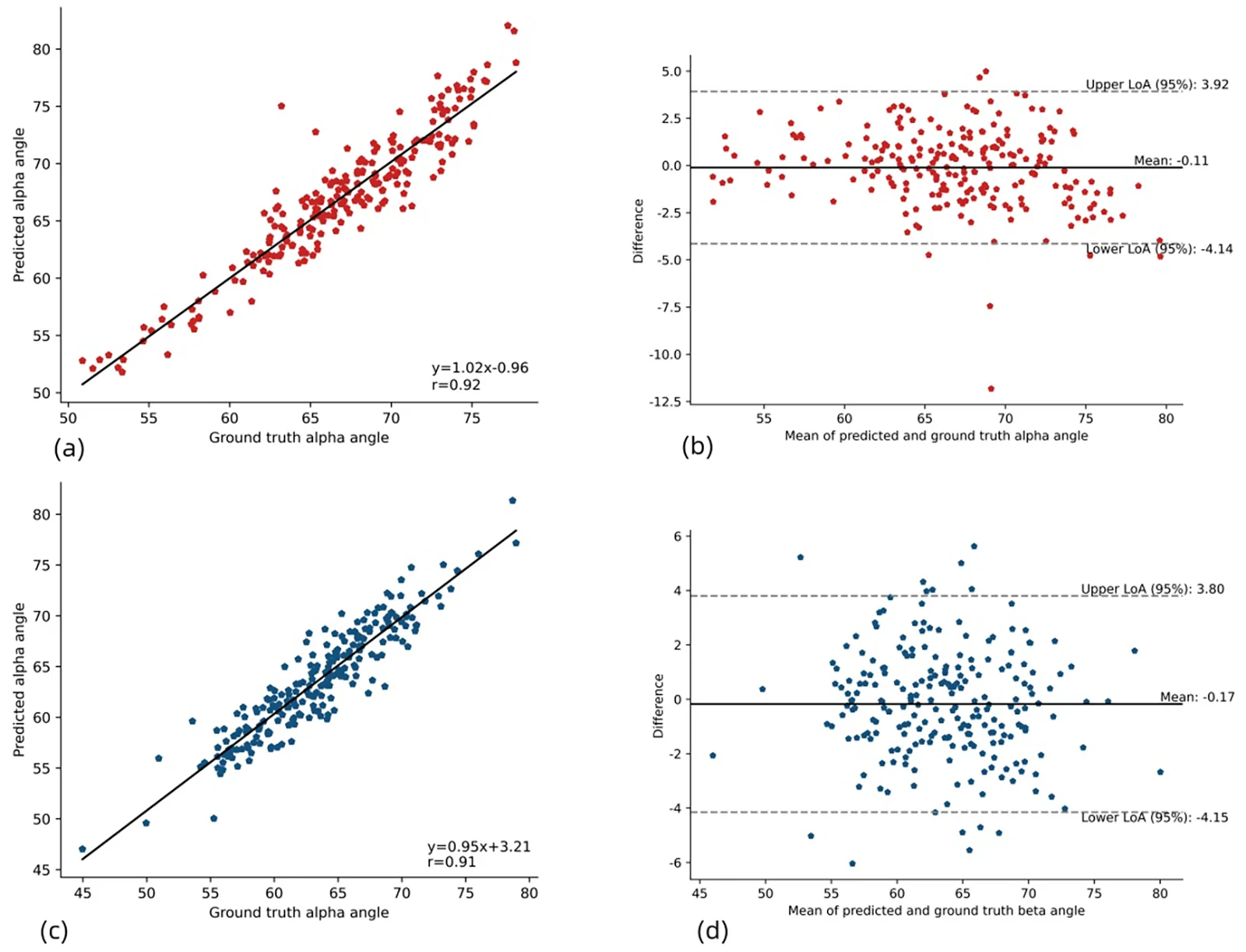
Scatter and bland–altman plots of the predicted and reference α and β angles. **(a)** and **(b)** compare true and predicted *α* angles; **(c)** and **(d)** compare true and predicted β angles. In the Bland–Altman panels **(b,d)**, the solid line is the mean difference (bias) between predicted and reference angles and the dashed lines are the 95% limits of agreement (bias ± 1.96 SD); the diagonal in the scatter panels **(a,c)** is the line of identity. Angles are in degrees and analysis is on the Dataset A test folds. *α* and β, Graf angles.

For the automated Graf classification, MultiHipPoseNet had mean angle errors of 1.662° and 1.756° and accuracy, precision, and recall of 97.79%, 92.11%, and 88.46%, respectively. Relative to the two ablations trained under the same protocol, the full model had the highest accuracy and precision and matched or exceeded their recall. The published values marked † are not directly comparable. The analysis included 200 type I and 26 type II images per test fold and no type III or type IV images.

### 3.4 Robustness of MT-GALM compared with hard parameter sharing

As shown in Figure 8, at the lowest PSNR the mean absolute errors of α and β for MultiHipPoseNet were 2.764° and 2.292°, respectively. Across the six PSNR levels, both angle errors were lower for MultiHipPoseNet than for MultiHipPoseNet−, and the between-model difference increased as PSNR decreased. The corresponding MIoU values show that MultiHipPoseNet had a higher MIoU at every PSNR level, with a larger absolute difference at lower PSNR (Figure 9).

**Figure 8:**
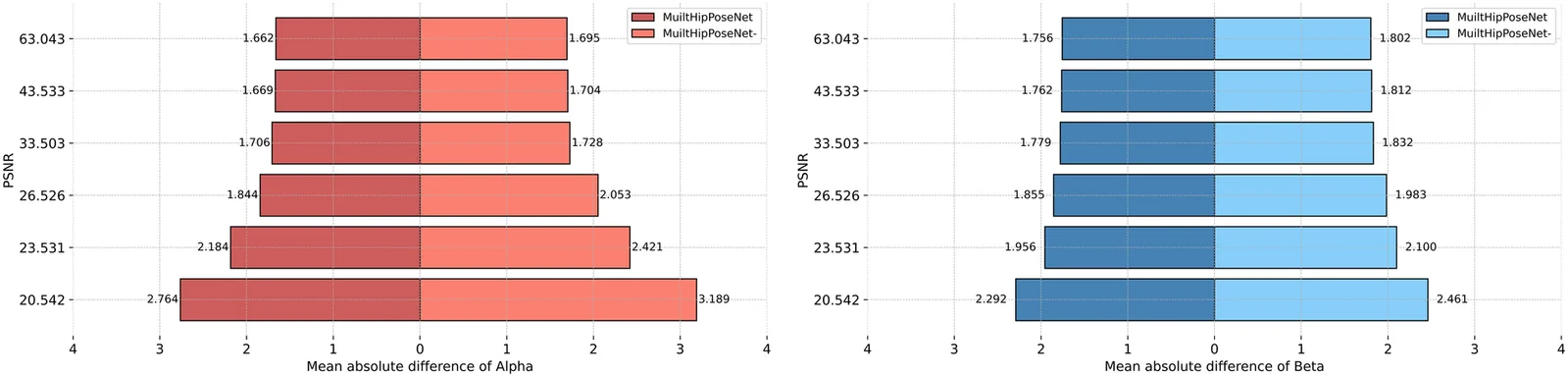
Predicted mean absolute errors of multiHipPoseNet and multiHipPoseNet− for the α and β angles on images with different PSNRs. Higher PSNR indicates better image quality, and lower mean absolute error indicates better performance. MAE, mean absolute error (degrees); PSNR, peak signal-to-noise ratio (dB), where lower PSNR denotes stronger salt-and-pepper noise; evaluated on the Dataset A test images. MultiHipPoseNet− is the ablation with ME-GCT but without MT-GALM.

**Figure 9:**
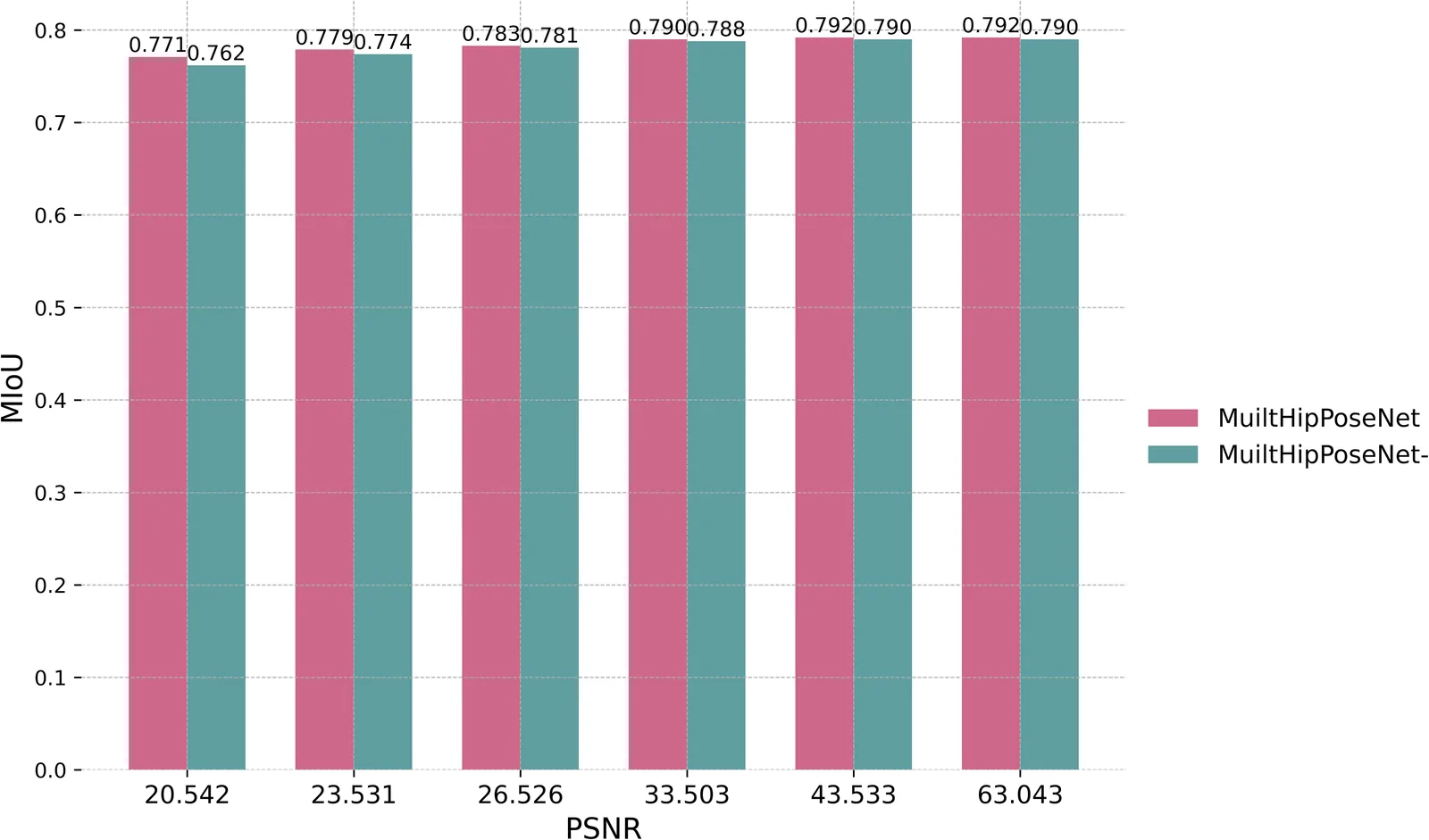
Segmentation performance of multiHipPoseNet and multiHipPoseNet− on images with different PSNRs. Higher PSNR indicates better image quality, and higher MIoU indicates better performance. MIoU, mean intersection-over-union; PSNR, peak signal-to-noise ratio (dB). Higher MIoU and higher PSNR indicate better segmentation and better image quality, respectively. MultiHipPoseNet− omits MT-GALM.

### 3.5 Performance across image qualities, hip types, and machines

The quality-stratified results for the machine-1 test images (illustrated in Figure 10) are reported in Table 5. For both methods, the error metrics were lowest in the good-quality category and highest in the poor-quality category. MultiHipPoseNet had higher DSC and lower HD, *Δ*D, α-angle error, and β-angle error than HipPoseNet in each of the three quality categories.

**Figure 10:**
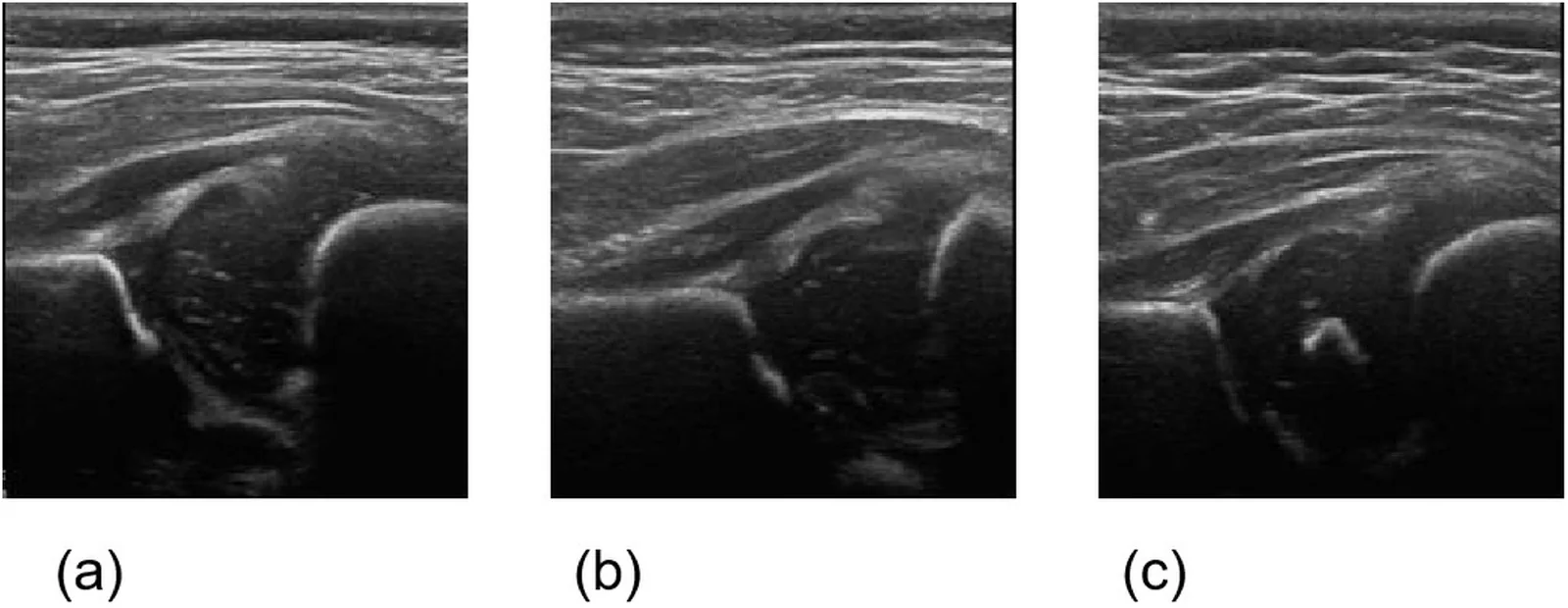
Three DDH images of different quality. **(a)** good quality; **(b)** medium quality with blurred edges; **(c)** poor quality with invisible structures. Image quality was graded as good (structures and interfaces clearly visible), medium (blurred edges), or poor (one or more target structures poorly visible). DDH, developmental dysplasia of the hip.

**Table 5:** Test performance of multiHipPoseNet and hipPoseNet across three image qualities.

Method	Image quality	DSC (avg)	HD (avg)	*Δ*D (avg)	α (°)	β (°)
HipPoseNet	good	0.861	8.252	0.679	1.732	1.994
HipPoseNet	medium	0.854	8.677	0.718	1.998	2.141
HipPoseNet	poor	0.844	9.876	0.892	2.399	2.481
MultiHipPoseNet	good	**0**.**869**	**7**.**688**	**0**.**602**	**1**.**401**	**1**.**550**
MultiHipPoseNet	medium	**0**.**863**	**8**.**086**	**0**.**631**	**1**.**588**	**1**.**709**
MultiHipPoseNet	poor	**0**.**852**	**8**.**819**	**0**.**761**	**2**.**034**	**2**.**035**

DSC and HD are segmentation metrics; *Δ*D is the mean landmark error (mm); α and β are angle errors (°). DSC, Dice similarity coefficient; HD, Hausdorff distance (pixels); *Δ*D, mean Euclidean landmark error (mm); α and β, mean absolute angle errors (degrees). Results are for the Dataset A (machine 1) test images stratified into good, medium, and poor quality.

As shown in Table 6, DSC and HD were similar for type I and type II images (0.861 vs. 0.860 and 8.188 vs. 8.100, respectively). Type II images had a lower mean landmark error (0.609 vs. 0.668 mm) and lower *α*-angle error (1.359° vs. 1.702°), but a higher β-angle error (2.124° vs. 1.708°). These subgroup results were summarized descriptively.

**Table 6:** Test performance for type I and type II hips.

Hip type	DSC (avg)	HD (avg)	*Δ*D (avg)	α (°)	β (°)
Type I	0.861	8.188	0.668	1.702	1.708
Type II	0.860	8.100	0.609	1.359	2.124

DSC and HD are segmentation metrics; *Δ*D is the mean landmark error (mm); α and β are angle errors (°). Metrics are defined as in Table 5. Results are descriptive summaries for Graf type I and type II hips in the Dataset A test folds (200 type I and 26 type II images per fold); no formal type I-versus-type II hypothesis test was performed.

On the held-out Dataset B from machine 2 (Figure 11), MultiHipPoseNet achieved DSC 0.861, HD 8.100 pixels, mean landmark error 0.769 mm, α-angle error 2.306°, and β-angle error 2.678° (Table 7). These values were higher for DSC and lower for the four error metrics than the corresponding HipPoseNet values.

**Figure 11:**
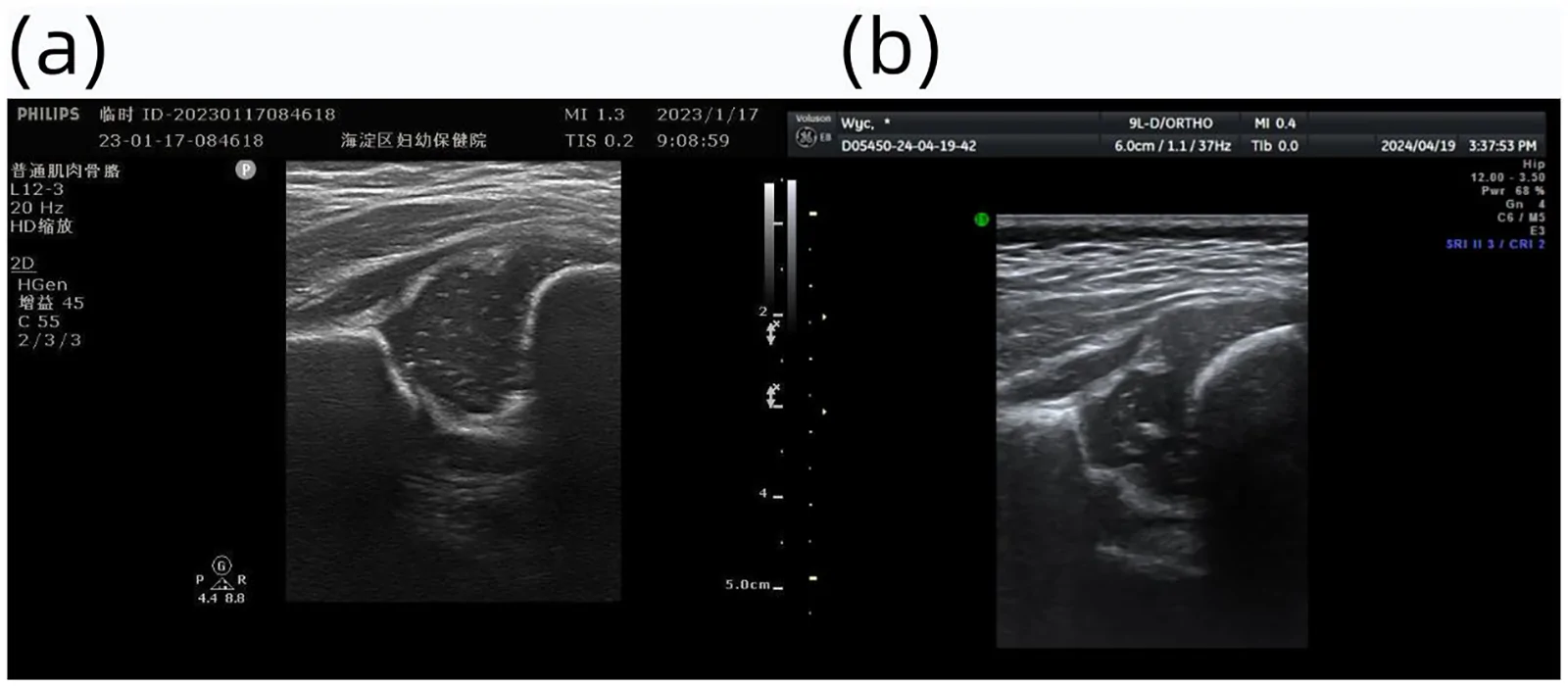
Typical ultrasound images from the two machines. **(a)** image from machine 1; **(b)** image from machine 2. Screen-header metadata were masked to ensure de-identification; the ultrasound fields were not altered. Machine 1 corresponds to the development set (Dataset A) and machine 2 to the external test set (Dataset B).

**Table 7:** Test performance of the methods evaluated on external dataset B (machine 2).

Method	DSC	HD	*Δ*D	α (°)	β (°)
U-Net	0.837	10.865	–	–	–
HipPoseNet	0.826	8.242	0.892	2.577	2.853
MultiHipPoseNet	**0**.**861**	**8**.**100**	**0.769**	**2.306**	**2.678**

DSC and HD are segmentation metrics; *Δ*D is the mean landmark error (mm); α and β are angle errors (°). Bold indicates the best result. Metrics are defined as in Table 5. Results are for the external Dataset B (machine 2; 226 images from 96 infants), which was not used during training. Dashes denote metrics not computed (landmark and angle errors are undefined for the segmentation-only U-Net baseline).

## 4 Discussion

To address the limitations of existing deep-learning methods in fully considering structural information and in accounting for the standard plane, this work proposes a multi-task network, MultiHipPoseNet, for DDH analysis and diagnosis that marks anatomical structures and performs automatic angle measurement. Experimental results show that our method makes full use of the structural information in hip images while respecting the role of the standard plane, and learns the interaction between structures and landmarks with good accuracy. Compared with previous approaches that obtain anatomical boundaries or indirectly infer landmark coordinates from structural features (20–22), directly predict landmark coordinates (23, 24), or perform radiograph-based automated measurement and morphological classification (25, 27), our method jointly models seven anatomical structures and six landmarks in hip ultrasound while retaining standard-plane assessment. The landmark coordinates are predicted in one step without neglecting the standard plane, and the method does not overfit the parameters of a traditional classification network when diagnosing DDH, offering good interpretability.

The agreement between predicted and reference angles also improves on prior work. Golan et al. (20) reported an α-angle correlation of 0.76, and Lee et al. (21) reported correlations of 0.764 and 0.743 for the α and β angles. In comparison, our method attains Pearson correlations of 0.92 and 0.91 for the α and β angles, an improvement of approximately 0.16 for α and 0.17 for β over these landmark-based baselines, which we attribute to the joint modelling of structures and landmarks and to the cross-task dependencies captured by MT-GALM.

The ablation results are consistent with complementary contributions from ME-GCT and MT-GALM: the full model improved landmark SDR at all three thresholds and improved most structure-specific HD values relative to the ME-GCT-only variant. The larger horizontal error at the baseline point is unlikely to affect the Graf angles materially because the annotation protocol emphasizes the vertical positions of the ilium endpoints. The larger POAP error and the disrupted cartilage example in Figure 6 may reflect weak boundaries, echo attenuation, structural deformation, or off-axis acquisition. The noise experiments further suggest that modelling cross-task dependencies is less sensitive than hard parameter sharing as PSNR decreases.

The type-specific results require cautious interpretation. Type II images had lower mean landmark and *α*-angle errors but higher β-angle error than type I images; because only 26 type II images were available per fold, this pattern may be unstable and was therefore treated descriptively. Likewise, the published values of Hu et al., Xu et al., and Chen et al. were obtained on different datasets and cannot support a claim of superiority in a like-for-like comparison. Finally, no reader study was performed on the same held-out test set, so the 5° success threshold must not be interpreted as evidence that the model is equivalent to a professional sonographer.

Our method has several limitations. In some images of hip dysplasia, key structures such as the cartilage may be obscured by structural deformation or filming angle, which can cause the predicted segmentation mask to fail. More importantly, because of the insufficient number of positive cases (types III and IV), our method was validated only on type I and type II hips; since the clinical purpose of DDH screening is to detect abnormal and dislocated hips, the present results demonstrate technical feasibility on normal and mildly dysplastic hips rather than established clinical utility, and should not be over-interpreted as a validated clinical decision-support tool. Given storage and computational constraints (57), fast and effective two-dimensional hip sonography is currently preferred, particularly in low-resource settings; nevertheless, developing accurate three-dimensional DDH analysis is valuable. We will therefore continue to collect more hip cases, including type III and type IV hips, to extend the method to all hip conditions, and will investigate three-dimensional DDH analysis.

## 5 Conclusion

In this study, we propose a comprehensive multi-task learning framework for automatic and accurate angle measurement while marking anatomical structures. It uses ME-GCT to optimize feature extraction, explicitly modelling the relationships between tasks and channels to balance collaboration and conflict across tasks. It further leverages MT-GALM to mine the hidden dynamic dependencies between adjacent channels across tasks, reducing the risk of overfitting to a single task and improving robustness.

We evaluate the method in three ways. First, landmark detection and structure segmentation are assessed through comparative and ablation experiments. Second, angle prediction and the classification of type I and type II hips are evaluated through post-processing, with internal ablation comparisons and contextual reporting of published methods. Third, we verify the robustness of the multi-task graph adaptive learning mechanism against hard parameter sharing, and test generalization across image qualities, hip types, and machines. The results show average errors of 1.662° and 1.756° for the α and β angles, with 97.97% and 96.81% of estimates within 5°, and classification accuracy, precision, and recall of 97.79%, 92.11%, and 88.46% for hip type. Because validation was limited to type I and type II hips, these findings establish technical feasibility on normal and mildly dysplastic hips; evaluation on type III and type IV hips is required before clinical adoption.

## Data Availability

The raw data supporting the conclusions of this article will be made available by the authors, without undue reservation.
